# Cognitive impairment in a young marmoset reveals lateral ventriculomegaly and a mild hippocampal atrophy: a case report

**DOI:** 10.1038/srep16046

**Published:** 2015-11-03

**Authors:** A. Sadoun, K. Strelnikov, E. Bonté, C. Fonta, P. Girard

**Affiliations:** 1 Université de Toulouse; UPS; Centre de Recherche Cerveau et Cognition; Toulouse, France; 2 CerCo, CNRS UMR 5549, Toulouse, France; 3 INSERM, Toulouse, France

## Abstract

The number of studies that use the common marmoset (*Callithrix jacchus*) in various fields of neurosciences is increasing dramatically. In general, animals enter the study when their health status is considered satisfactory on the basis of classical clinical investigations. In behavioral studies, variations of score between individuals are frequently observed, some of them being considered as poor performers or outliers. Experimenters rarely consider the fact that it could be related to some brain anomaly. This raises the important issue of the reliability of such classical behavioral approaches without using complementary imaging, especially in animals lacking striking external clinical signs. Here we report the case of a young marmoset which presented a set of cognitive impairments in two different tasks compared to other age-matched animals. Brain imaging revealed a patent right lateral ventricular enlargement with a mild hippocampal atrophy. This abnormality could explain the cognitive impairments of this animal. Such a case points to the importance of complementing behavioral studies by imaging explorations to avoid experimental bias.

For many decades, neuroscientists have used different animal species as models to study various aspects of cognition and learning. In most cases, those animals were considered to be healthy on the basis of classical veterinary investigations. Thus, most studies were conducted classically without considering the possibility of brain anomalies, especially in the case of outliers, where animals appeared to be low performers. The latter are generally rightly excluded on the basis of the experimental conditions, such as social interactions^1^ or kept in experiments with a large number of subjects. Nevertheless, in some studies, these outliers could be excluded without seeking further explanations, especially pathological ones. On the other hand, using imaging may lead to the fortuitous discovery of unexpected silent brain anomalies. This often happens with the increasing use of imaging in humans, raising the idea that unexpected brain pathologies are not as rare as one might think^2^. In this context, several types of anomalies can be discovered^3^,^4^ such as brain neoformations, prominence of the subarachnoid spaces, vascular malformations, and dilatation of the ventricular system.

Ventricular enlargement or ventriculomegaly is the most common brain anomaly observed in fetal ultrasound images^5^. Some authors using MRI observed the existence of this anomaly in experimental animals, although there was no external signs^6^. In MRI studies, it can manifest itself as an enlargement of the whole lateral ventricle or be limited to one of it parts, such as the temporal ventricular horn^7^. This anomaly persists frequently into adulthood^5^. Because of the complexity of the ventricular anatomy, especially the lateral ventricles, and the fact that ventriculomegaly is being linked to pathological processes related to atrophy, dysgenetic anomalies or obstructive hydrocephalus^8^,^9^, it is often associated with an impairment of the adjacent brain structures such as the hippocampus, the amygdala, the caudate nucleus and the periventricular white*-*matter fibre bundles^10^. Subjects who show this pathology present with a pattern of neurological impairments or a set of subtle cognitive anomalies^11^.

In animals, several cases of ventricular enlargement were used as models related to mental retardation with genetic etiology^12^, psychotic disease^13^ and sublethal hypoxia^14^. Likewise, some authors^6^, using rats purchased from two different companies as an animal model of brain traumatic injury, discovered that nearly half (43.2%) of animals had spontaneous ventriculomegaly without external manifestations. In their study, they pointed out the necessity of using imaging explorations to avoid a high degree of variability that can be the result of the ventricular enlargement itself. In non human primates, ventricular enlargement has not yet been reported to the best of our knowledge.

In a behavioral study assessing cognitive skills in marmosets where structural MRI imaging was scheduled, we report a young female marmoset which required significantly more time than other animals to learn two different behavioral tasks (shape discrimination and delayed matching-to-position). This animal showed a range of cognitive anomalies concerning the behavioral strategies it used to perform these tasks. MRI investigations revealed the existence of a striking right ventricular horn enlargement with a mild hippocampal atrophy in that animal. This raises a crucial issue for neurobiological and behavioral studies concerning the necessity of using imaging to exclude brain anomalies in the case of outliers or poorly performing individuals.

## Results

Data analysis showed a striking set of cognitive impairments for the pathological case (PathC) related to the latency to reach the learning criterion in both tasks in addition to several anomalies concerning the ability to apply appropriate learning strategies.

All data are expressed in *mean* ± *SEM*.

*A- Behavioral data*

*1- Simple Discrimination Task (SD):*

**Figure 1:**
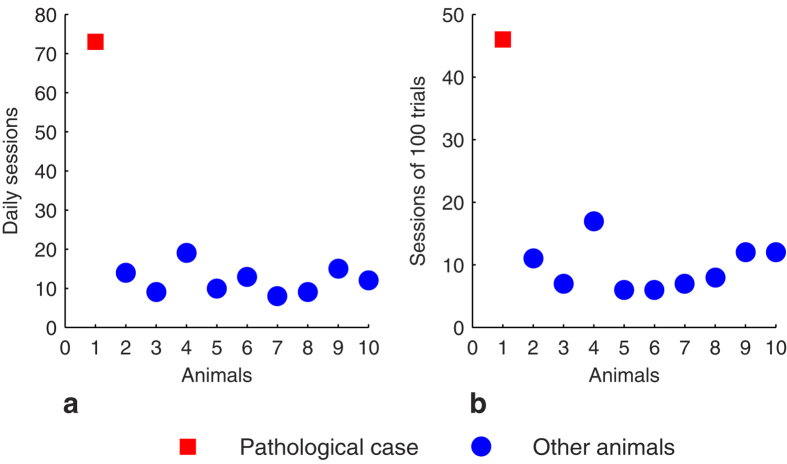
Learning performances in the SD task. Number of daily sessions (**a**) and those of 100 trials (**b**) necessary to reach the learning criterion.

All marmosets, except PathC, performed this task and reached the criterion of learning in 9.5 ± 1.2 sessions of 100 trials, corresponding to 12.1 ± 1.1 daily sessions. This contrasts with the marmoset PathC which took significantly more sessions and more time to reach the learning criterion in this task (Fig. 1a,b): 46 sessions of 100 trials corresponding to 73 daily sessions. The confidence intervals (CI) of the other animals at *P* < *0.05* did not include the PathC’s values (*CI* = *[7.4 12.8]* for the number of sessions of 100 trials and *CI* = *[10 15.1]* for the number of daily sessions).

### Response strategies analysis

In order to analyse the response and learning strategy features in this task, we divided it into two phases: the learning (before reaching the criterion) and the learnt phases.

**Figure 2:**
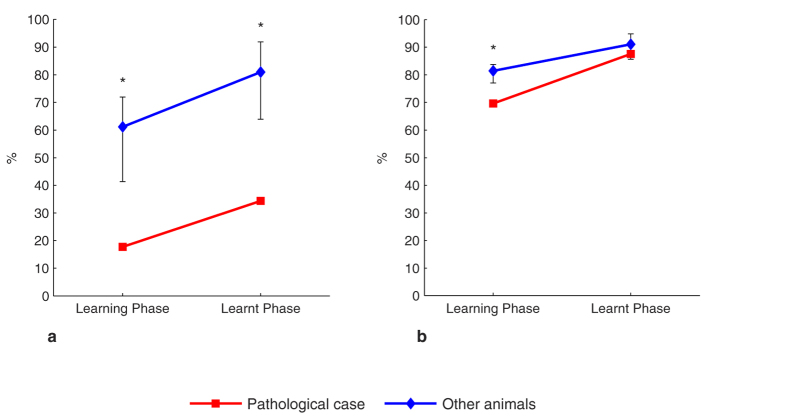
Response strategies in the SD task. The occurrence of the LSh (**a)** and the ChSh sub-strategies (**b**) in the learning and learnt phases. Error bars represent the 95% bias-corrected and accelerated bootstrap confidence intervals (*P* < *0.05*). *Significant.

In the learning phase, the PathC showed a statistically significant difference in applying the Lose-Shift (LSh) (17% for the PathC, whereas the *CI* of the other animals was *[41*, *71]*) (Fig. 2a) and the Change-Shift (ChSh) sub-strategies (69% for the PathC, whereas the *CI* of the other animals was *[77*, *83]*) (Fig. 2b). However, there was no significant difference between our case (90%) and the other animals (*CI* = *[88*, *92]*) concerning the Win-Stay (WSt) sub-strategy.

In the learnt phase, there was no significant difference (*P* > *0.05*) between the PathC and the other animals concerning the WSt and the ChSh strategies. Nevertheless, for the latter strategy, the PathC showed a lower value (87.6%) than the other animals with a medium effect size (*Cohen’s d* = *0.62*). (Fig. 2b). Also, LSh sub-strategy occurred significantly less (34%) for the PathC than the other animals (*CI* = *[63*, *91]*). (Fig. 2a).

*2 – Delayed Matching-To-Position task (DMTP)*

All animals reached the learning criterion. The animals performed an average of 22 ± 1.8 sessions of 100 trials corresponding to 27.5 ± 3.3 daily sessions.

**Figure 3:**
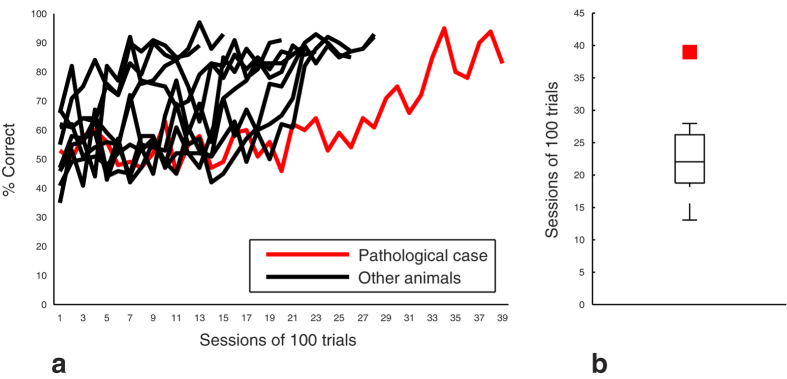
Learning performances in the DMTP task. (**a**) Individual learning curves. Data were calculated after discarding correction trials (see methods). (**b**) Number of sessions to reach the learning criterion. Data are represented as median and interquartile intervals. The red square represents the performance of the PathC.

The PathC required more sessions (39 sessions of 100 trials) (Fig. 3) and more time (59 daily sessions) to reach the criterion in this task. Statistical analysis showed that the animals’ *CI* at *P* < *0.006* did not include the PathC’s values concerning the number of sessions of 100 trials and daily sessions (respectively *CI* = *[16.7 26.1]*, *[19.4 35.8]*). None of the animals presented obvious position habits that could have helped them to solve the task.

We divided this learning phase in three equal blocks of trials for each animal and calculated the mean percent correct responses in those blocks for both the PathC and the other animals. Statistical analysis did not show any difference between performances although PathC showed a tendency to have lower mean correct responses across the blocks (*d* = *0.37* and *d* = *0.76* for the last two blocks respectively).

Concerning the longer delays (3 to 12 sec), the percentage of correct responses decreased gradually with delay length for all the animals. Significant differences appeared for 3 and 9 sec delays where PathC showed better performances than the other animals.

The next phase of the task consisted in the randomized-delays step, divided into four equal blocks of 300 trials each. In this case, data showed a gradual improvement of accuracy for the other animals contrasting with the PathC which showed lower performances than other animals with a medium (*d* = *0.43*) and large (*d* = *1.05*) effect size in block 2 and 3 respectively. Moreover, in block 4, a significant statistical difference was found between the PathC (74%) and the other animals (*CI* = *[80.7 87.3]*). The PathC did not have a stable gradual improvement.

### Response strategies analysis

#### Learning phase

**Figure 4:**
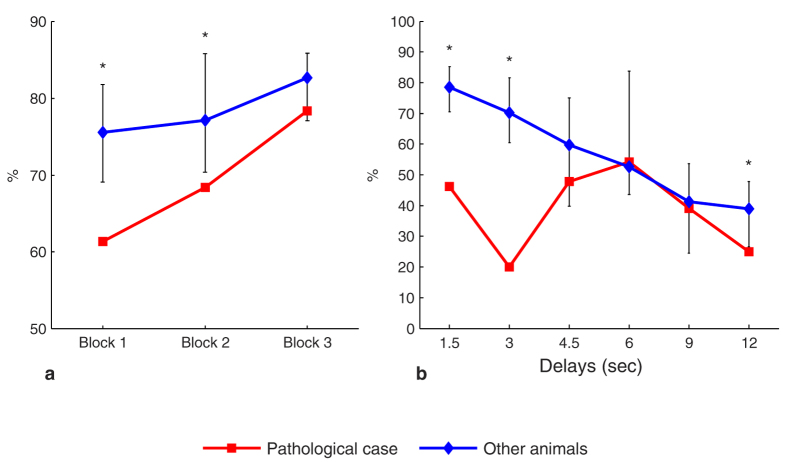
Evolution of the animals’ response strategies in the DMTP task. (**a**) The Win-Stay sub-strategy in the learning phase. (**b**) The Lose-Shift sub-strategy in the DMTP task as a function of the delay. Error bars represent the bias-corrected and accelerated bootstrap confidence intervals (*P* < *0.006*). *Significant.

We divided this phase into three equal blocks and computed the occurrence of each strategy in each block for the PathC and the other animals. The main result concerns the WSt sub-strategy which occurred at a higher level for the other animals in block 1 (*CI* = *[69*, *80]* at *P* < *0.006*) compared to the PathC which showed a significantly lower occurrence in this block (61%), and also a significantly lower value in block 2 (68%) in comparison to the other animals (*CI* = *[70*, *85]*). Block 3 showed a strong tendency to lower values with a large effect size (*d* = *0.99*) for the PathC without a significant difference compared to the other animals (Fig. 4a). For the other sub-strategies, PathC showed a tendency to obtain lower values, mainly in Block 2 and 3 compared to the other subjects. Indeed, concerning the LSh sub-strategy, a tendency to lower values was shown for the PathC in the last two blocs, with small (*d* = *0.32*) and medium (*d* = *0.62*) effect size respectively. Likewise, there was a tendency to lower occurrence of the ChSh strategy for the PathC with a medium effect size across the blocks (Respectively: *d* = *0.62*, *d* = *0.64*, *d* = *0.58*).

#### Learnt phase

No significant difference was seen concerning the ChSh and the WSt sub-strategies. Nevertheless, for the latter sub-strategy, as in the learning phase, PathC kept its tendency to have a lower value with a large effect size (*d* = *1.0087*). However, data showed a significantly lower occurrence (46%) of the LSh sub-strategy for the PathC at *P* < *0.006* compared to the other animals (*CI* = [70, 85]).

#### Non randomized-delays step

Data showed mainly significant differences (*P* < *0.006*) concerning the shift strategies. For some delays, the PathC case adopted a ChSh sub-strategy, which occurred at higher and significant frequencies compared to the other animals. This result contrasts with the LSh sub-strategy which occurred for some delays at significantly lower values for the PathC (Fig. 4b).

#### Randomized-delays step

**Figure 5:**
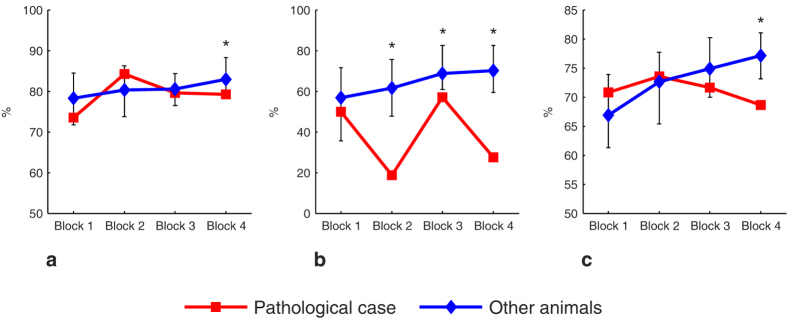
Evolution of the response strategies in the randomized-delay step in the DMTP task. **(a)** WSt sub-strategy. **(b)** LSh sub-strategy. **(c)** ChSh strategy. Error bars represent the bias-corrected and accelerated bootstrap confidence intervals (*P* < *0.006*). *Significant.

In this step, the occurrence values of all the strategies gradually increased across the block sessions for all other subjects. This improvement contrasts with those of the PathC which showed an unstable strategy in performing this task at this step (Fig. 5).

*B- MRI*

Eight animals underwent MRI imaging.

#### Manual measurements

**Figure 6:**
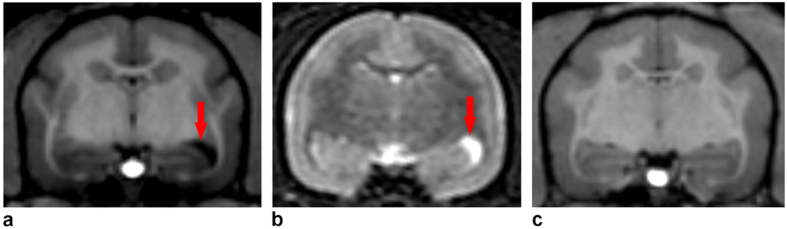
MRI images. MRI T1 and T2 weighted scans showing the contrast between the abnormal ventricular enlargement (arrow) in the PathC (**a,b**) and another animal (**c**)

The volume of the right temporal ventricular horn of the other animals was 0.98 ± 0.56 mm^3^ while the PathC showed a striking enlargement of this structure (8.13 mm^3^) (Fig. 6). This value was statistically different from those of the other subjects (*CI* = *[0.30 2.69]* at *P* < *0.05*).

Additionally, we performed another MRI scan one year later for the PathC and found no striking evolution of the ventricular enlargement (8.53 mm^3^).

Likewise, the volume of the right hippocampus (RH) at the region of interest (ROI) of the other animals was different (14.34 ± 0.03 mm^3^) from that of the pathological case (14.07 mm^3^). This difference was statistically significant (Animals’ *CI* = *[14.28 14.42]* at *P* < *0.05*).

#### Automated measurements

Concerning the ventricular volumes, we could not perform an automated segmentation because of the variability of the temporal ventricular horn position. Thus, we focused the automated measurements on the volume of the RH in the ROI.

The animals’ volume of the RH at the ROI was 14.97 ± 0.24 mm^3^ while that of the pathological case was 13.23 mm^3^. This difference was statistically significant (animals’ *CI* = *[14.49 15.48]* at *P* < *0.05*).

Likewise, we measured the difference of the obtained volumes between the manual and the automated segmentation. Those differences were close to 0 (*CI* = *[0.12 1.04]* indicating that the differences were about the size of one voxel.

## Discussion

We report a pathological case that presented behavioral impairments in two tasks, a spatial and a non-spatial one. Data analysis showed a range of cognitive anomalies for the PathC related to the latency to reach the learning criterion in both tasks. This delay in learning is associated with several problems related to the ability to apply appropriate learning strategies.

The impairments were associated with a striking lateral right ventricular enlargement of the temporal horn. Data analysis showed a significant difference between the right temporal ventricular horn volume which was increased in the PathC relative to that of the other animals. Moreover, with two types of measurement, a manual and an automated one, we found a significantly lower volume of the right hippocampus part close to the ventricular dilation, in comparison to that of the other animals.

In humans, lateral ventricular enlargement is involved in a huge range of pathologies such as schizophrenia^13^,^15^, bipolar disorders^15^,^16^, and some clinical forms of Parkinson’s disease^17^.

The ventricular enlargement is underlain by a pathological mechanism related to the atrophy of brain tissues (passive mechanism) or to other active processes leading to the damage of periventricular structures^8^,^9^. Regarding our MRI analysis, we performed two T1 weighted MRI scans one year apart for the PathC and did not see a patent evolution of the ventriculomegaly volume. This result is in line with a passive process related to an atrophic mechanism. As a consequence, ventricular enlargement leads to several substantial neurological and cognitive manifestations as mentioned above or subtle signs related to cognitive deficits such as working memory impairment and psychological disabilities that only a large set of neuropsychological investigations can detect or assess^11^,^18^.

In our study, two tasks were used for assessing cognitive skills in marmosets, the SD and the DMTP tasks. To solve them, an animal needs not only forming a stimulus-reward association, but also acquiring a learning set that requires transferring a set of information to a new case in the same problem space^19^. This corresponds to the entire probable configurations that a given problem can take in a task. Thus, for the simple discrimination task, after acquiring a learning set, an animal should have full proficiency with a new pair of stimuli. According to Harlow’s model^20^, solving the simple discrimination problem implies identifying the pair of stimuli as the problem space and recognizing the relevant parameters (shape, texture) rather than their locations on the screen to differentiate them. Furthermore, resolving the DMTP task requires identifying the relevant location during the choice phase according to that of the sample phase. Thus, this task implies the use of working memory for the retention of the information during the delay period to recall it in the choice phase and update it in the subsequent trial.

Likewise, learning sets involve the use of the WSt-LSh rule^21^, a natural strategy for the marmoset^22^, which implies keeping the information of the correctness or incorrectness of the previous trial in order to use it in the next one^23^. Besides, as applied here, the ChSh strategy requires shifting the response in the subsequent trial when the location of the rewarded stimulus or that of the sample one in the DMTP task changes, reflecting the adaptive ability of a subject to avoid non-relevant information.

Little is known about the neural basis of the response strategies used for resolving a given problem in a cognitive task^24^,^25^. However, a number of studies have pointed out the involvement of some key structures such as the hippocampal system^26^,^27^,^28^, the caudate nucleus^27^, the prefrontal cortex^29^,^30^,^31^ and the putamen^32^.

In our study, we showed that the PathC had a marked delay in learning both tasks. This deficit was characterized by an impairment in applying the adapted strategy, only displayed by that subject. In the SD task, we noticed a striking and statistically significant lower occurrence for LSh and ChSh strategies in the learning phase followed by an improvement in the learnt phase for the latter strategy, but without a strong increase in the occurrence of the LSh sub-strategy.

The deficit in using the LSh sub-strategy may rely on a lower reward value sensibility which may be linked to an impairment in the amygdala^33^,^34^,^35^, a brain structure involved in the association between the environmental stimuli and whether or not an experience is rewarded, and which contributes to prefrontal reward value encoding^36^,^37^. Furthermore, other studies highlighted the role of various structures involved in reward processing: the ventral and dorsal striatal system^38^,^39^,^40^,^41^, including caudate nucleus^42^,^43^ and orbitofrontal cortex^44^,^45^. Some authors^27^,^43^,^46^,^47^ pointed out the role of the caudate nucleus in reward and value-based choices either in shift or stay responses. Nevertheless, some authors^27^,^47^ argued that the caudate nucleus is implicated in a stay behavior, mainly in a WSt task, and on the contrary, the hippocampal system is involved in a shift strategy^27^. More recently, other authors^48^ showed a lower probability of shifting choices after error responses in rats with striatal lesions, mostly dorsolateral.

Since rats naturally use a win-shift/lose-stay strategy in foraging^49^, contrary to marmosets which naturally show a WSt/LSh strategy^22^, we suggest that the role of these aforementioned structures regarding response strategies should also be interpreted according to the ecological behavior of the species that is used in an experimental context. Interestingly, some authors, in an earlier study^50^, performing lesions on different parts of the caudate nucleus in monkeys saw a higher error rate before reaching the learning criterion in the object visual discrimination task concerning the group with lesions on the tail of the caudate nucleus. This result is similar to those observed after lesions in the inferotemporal cortex which sends projections to the tail of the caudate nucleus^50^,^51^. Because this region of the caudate nucleus is anatomically located near the temporal ventricular horn, an anomaly of this ventricular structure also leads to an impairment of the tail of the caudate nucleus. In the MRI of PathC, we found two major anatomical anomalies, a striking unilateral enlargement of the right temporal ventricular horn and a mild hippocampal atrophy. However, we could neither evaluate the volumes of the caudate nucleus and the amygdala near the enlarged ventricle nor the size or the thickness of the periventricular white*-*matter fiber bundles. Thus, we cannot rule out the possibility of an impairment of these structures near the abnormally enlarged ventricular horn in PathC.

In regard to the other strategies, the normal subjects used both the ChSh and WSt at roughly the same occurrence. However, the PathC had a lower use of the ChSh strategy in the learning phase, whose occurrence improved in the learnt phase of the SD task. This latter feature may, according to some authors, be related to the hippocampal damage^28^. Nevertheless, these authors assessed a higher level of information processing than in our tasks where ChSh concerns the change of the relevant position across trials. The weakness of using this strategy by the PathC appears to be a difficulty in shifting from the previous location to another across trials. This is compatible with a response perseveration that may be explained by a hippocampal impairment^52^,^53^,^54^.

In the learning phase of the DMTP task, we saw a significantly lower occurrence of the WSt substrategy in the first and second blocks, and a strong tendency to a lower value in the last one. In the learnt phase, the PathC had, however, improved its performance by using this strategy. Since the WSt strategy was not affected in the SD task, the delay component of the DMTP task may result in an occasional failure of working memory even if the delay in the beginning stage of this task was short. Besides that, no significant difference was observed between the PathC and the other marmosets during the learning phase concerning the ChSh strategy across blocks. Since the use of this strategy was improved by the PathC in the later stage of the SD task and concerns the relevant location in our conditions in both tasks, we hypothesize, according to Harlow’s model, the existence of a learning transfer between the two tasks. In addition, during this learning phase, there was no difference in the proportion of LSh sub-strategy between the PathC and other marmosets. Since the latter sub-strategy concerns the location in the DMTP task (instead of the stimulus in the SD task) and also the fact that resolving this task relies on a different problem space, there was no reason to expect the same level of occurrence of this substrategy as in the SD task. However, in the learnt phase, the other animals improved significantly their use of this sub-strategy while the PathC did not. This abnormal feature was maintained across some longer delays, showing an unstable use of this sub-strategy rule by the PathC. The latter characteristic was also seen in the randomized-delay step. Likewise, in this step, that instability also concerned the other sub-strategies. The DMTP task requires prefrontal-hippocampus interactions involving working memory^55^. In PathC, the impairment concerns a single hippocampus. In such unilateral cases^56^ even if the lesion is partial^57^, one observes disturbance of the functioning of the contralateral one, hence causing an occasional failure of this memory^58^. This failure could result from events that disrupt the application of learning strategies^59^,^60^ and can be seen in a naturalistic-like approach^61^(for contrasting views see^62^,^63^). Thus, the integrity of these structures is fundamental for acquiring both SD and DMTP.

Focusing on the mean percent correct response in the non-randomized-delays step, performances decreased across longer delays to chance level at 12 sec for both the PathC and the other animals. However, when the delays were randomized, the other subjects showed a gradual and stable improvement in performance that was not seen in the PathC which showed a tendency to have a lower and unstable performance. This can be explained by the fact that the randomization of the delays puts a significant load on the working memory. Likewise, since the hippocampus is involved in much more than the spatial domain, the weak performances of the PathC may also rely on a slower learning of stimulus-response association related to an impairment of the hippocampal system^24^,^64^.

In summary, our study raises and highlights a number of key ideas. Firstly, the presence of an anatomical anomaly in radiological investigations should be considered in the case of an abnormal pattern of learning or performance in animals. In addition, our study points in the same direction as those studies that consider the hippocampus beyond its classical behavioral role^64^,^65^. Secondly, our analysis highlighted that some behavioral features reflecting an inability to use previously experienced situations to guide current choice were associated with anatomical anomalies. Ventriculomegaly, which can be caused by a range of etiologies, is indeed associated with several brain impairments^5^,^12^,^66^ and could explain the deficits we observed. However, because of the complexity of the learning process, involving many crucial areas and anatomical structures belonging to a complex network, we are aware that the ventriculomegaly may not be the only cause of the deficits we recorded. Nevertheless and finally, the existence of an outlier pattern of results in a cognitive task in a young adult animal without other objective explanation argues in favour of further radiological investigations in order to avoid experimental bias.

## Methods

### Animals

Ten young marmosets *(Callithrix jacchus)*, four females and six males (2–4.5 years old), were bred in our laboratory. In the rest of the text, one of these animals is designated as the pathological case (PathC), a 4 years old female. The behavioral testing was conducted in the home cage in a naturalistic-like context and approach. Each animal was isolated in the upper part of the cage only during its experimental session but stayed in visual, auditory and tactile contact with the congeners. Apart from that, animals were free ranging, unconstrained and without privation.

The body weight of PathC was 350g. This was in the range of the other marmosets ‘weights (Mean ± SEM: 344 g ± 13.4) and normal for marmosets at this age range^67^.

All experiments were carried out according to the National Committee for Ethical Reflection on Animal Testing. (authorization number: MP/03/76/11/12).

### Behavior

#### Animal training and stimuli

We used visual stimuli (3 × 3 cm, 17° of visual angle at a marmoset arm reach) of different geometric shapes filled with different gray-level textures. These stimuli were displayed on a touch screen fixed in front of the home cage (see supplementary information). We used visual gray-level stimuli rather than colorful ones as this is recommended to avoid possible discrimination bias related to different phenotypes of colour vision in the marmoset (as mentioned in^68^).

Training and testing were performed 3 to 5 days per week between 13:00 and 20:00. All marmosets were naïve to the tasks. They got familiarized with the setup by placing a piece of gingerbread on the screen and delivering a reward when the animal reached out to it. The size of the piece was gradually reduced until the animal touched a gray square displayed on the screen in absence of gingerbread.

Correct responses were rewarded with banana compote, yogurt, or mashed chickpeas (according to the day of the week) delivered by a peristaltic pump (0.1–0.2 ml) via a centrally-located licker above the holes in order to deliver the reward 10 cm inside the cage (see supplementary information). This led the animal to disengage itself from the screen to avoid position habits.

#### Simple Discrimination Task (SD)

This task consists in learning to discriminate the positive stimulus in each pair. Each member of the pair was pseudo-randomly displayed at 3.5 cm respectively to the right and to the left of the center of the screen and at the same height. The animals had to learn which stimulus was rewarded until they reached the learning criterion of 80% correct responses^69^. After reaching the criterion for the first pair, animals were trained to discriminate 2 to 10 other pairs. Here, the learning criterion should be stable in two consecutive sessions of 100 trials for one pair and kept for the next two consecutive sessions of a new pair.

The same pairs of stimuli were used for all the animals in the same order until reaching the learning criterion. There was no constraint in time or in the number of trials in a daily session. The daily session ended when the animal cumulated 25 ml of reward corresponding to its daily semiliquid diet or when it did not perform the task during at least 5 minutes. A given session of 100 trials could be continued over several consecutive days until completion.

All correct responses were rewarded and followed by displaying a black screen during an inter-trial interval (ITI) of 3 sec. Wrong responses were punished by displaying a blue screen during 5 sec followed by the ITI.

#### Delayed matching-to-position task (DMTP)

This task consisted of two steps. In the first one (the learning phase) and on each trial, a different stimulus was pseudo-randomly displayed in one of two locations on the screen (right or left). A 10.5 cm distance separated the two locations. We used the same type of stimuli as in the simple discrimination, but different exemplars (varied polygonal shapes and textures). The stimuli were different across trials adding up to a total of 271 exemplars. As soon as the animal touched the stimulus, a white screen was displayed during a delay (0.5 to 1 sec) after which it reappeared again in the same location. Step 2 consisted in displaying pseudo-randomly a stimulus on each trial. As soon as the animal touched it, a white screen was displayed during a delay of 1.5 sec, then the same stimulus reappeared in the two locations, at the right and at the left. The marmoset had to touch the stimulus which appeared in the same location as previously in the current trial to get a reward. Additionally, the stimuli were different between trials to reduce proactive interference. When the animal reached the learning criterion (80% of correct responses in two consecutive sessions of 100 trials and 80% of correct responses in two consecutive daily sessions) followed by a stable performance during 500 trials, we increased the delay every 2 sessions of 100 trials until reaching 12 sec (Delays were: 3, 4.5, 6, 9 and 12 sec). Thereafter, the delays were randomised during 1200 trials.

All correct responses were rewarded and followed by displaying a black screen during an ITI of 2 sec. Wrong responses were punished by displaying a blue screen during 6 sec followed by the ITI.

In addition, in the learning phase, when a marmoset showed a significant side bias (20 consecutive responses to one side), a correction procedure was applied: the correct stimulus was displayed on the non-preferred side until the animal had performed 5 consecutive correct responses.

#### Response and learning strategies

We analyzed the Win-Stay/Lose-Shift and Change-Shift strategies for both tasks. They reflect the ability of the animal to adapt its responses according to its own experience. According to the type of response, in the Win-Stay (WSt) sub-strategy, the animal repeated a correct previous response, whereas in the Lose-Shift (LSh) it did not repeat an incorrect previous response. The correct (or incorrect) responses are those whose location of the positive stimulus (in the SD task) or sample stimuli (in the DMTP task) did not change from one trial to the next (unchanged trials). The Change-Shift (ChSh) sub-strategy reflects the ability to shift from the previous response when that location changed from one trial to another (changed trials). The proportion of WSt was calculated as the proportion of stay responses after a correct one with reference to the total correct unchanged trials. That of LSh was calculated as the proportion of shift responses after an incorrect one with respect to the total incorrect unchanged trials. The proportion of ChSh was calculated as that of the correct change responses after a change trial with respect to the total change trials.

### Imaging

#### MRI acquisition and analysis

Animals were intramuscularly anesthetized with Alphaxalone, 1.85ml/kg (Alfaxan, 10mg/ml, Jurox, Worcestershire, UK). The average acquisition time was 45 min. SpO2, heart and respiratory rates were constantly monitored.

MRI scans were performed using a 3-T scanner (Achieva; Philips, Best, The Netherlands). High-resolution anatomical images were acquired using a 3-D T1-weighted sequence (slice thickness 0.35 mm, Reconstructed resolution: 0.31 × 0.31 × 0.35 mm, repetition time/echo time 10.5/4.7 ms, flip angle 8°). For the PathC we added a T2 weighted MRI scan.

We used MRICron software to change the format of the images from DICOM to NIFTI. NIFTI images were processed in SPM8 in order to rigidly align all brain images. Then, we adjusted the origin of NIFTI images below the knee of the corpus callosum and coregistered all the individual images to the template, adapted from Hikishima *et al.*^70^, using the automated tool in SPM8.

Using MatLab software (v R2013a) we decomposed 3D NITFI Images into 2D TIFF format. Since the ventriculomegaly is underlain by a pathological processes related to atrophy or other active processes leading to the destruction of periventricular structures^8^,^9^ where the higher rate of that atrophy corresponds in general to regions near the ventricular enlargement, we focused our analysis on this region. Thus, using GIMP 2.8 Software for manual segmentation, we manually created binary inclusive masks for each coronal slice with a region of interest (ROI) adjacent to the dilated portion of the right temporal ventricular horn of our PathC. This ROI comprised 5 slices in the right hippocampus (RH) at the same location in each animal. In addition, we obtained binary inclusive masks for each coronal slice of the animals’ right temporal ventricular horn. Thus, volume measurements were performed for each temporal ventricle horn and ROI of the RH. All the calculations were performed in the template space, which permitted us to factor out the individual variability in the total brain volume.

We also used an automated segmentation method for obtaining animals’ ROI masks (see supplementary information). We used the obtained binary images of inclusive masks for the ROI to calculate the RH volume in the ROI. As in the manual method, all the calculations were performed in the template space.

### Statistical analysis

Statistical analyses were performed with MatLab (R2013a).

The Kolmogorov–Smirnov test rejected the null hypothesis of normality for the distribution of the volumes of anatomical structures described above and the behavioral data at the significance level of *P* < *0.05*. Likewise, testing the assumption of normality with normal Q-Q plots, all the distributions exhibited considerable deviations from normality.

The comparisons of behavioral performances as well as the volumetric values between the other animals and our PathC were performed using a bootstrap method (re-sampling 10000 times) with a bias corrected and accelerated percentile algorithm for confidence intervals^71^. The difference was considered to be significant if the animals’ confidence intervals (*CI*) at *P* < *0.05* did not include the pathological case’s values. For the DMTP task we used Bonferroni correction for multiple comparisons, setting the significance level at *0.006* according to the number of conditions (Learning, non randomized delays and randomized delays).

## Additional Information

**How to cite this article**: Sadoun, A. *et al.* Cognitive impairment in a young marmoset reveals lateral ventriculomegaly and a mild hippocampal atrophy: a case report. *Sci. Rep.* **5**, 16046; doi: 10.1038/srep16046 (2015).

## Supplementary Material

Supplementary Information

## References

[b1] Fagot J. & Paleressompoulle D. Automatic testing of cognitive performance in baboons maintained in social groups. Behav. Res. Methods 41, 396–404 (2009). 19363180 10.3758/BRM.41.2.396

[b2] Piatt J. H. J. Unexpected findings on brain and spine imaging in children. Pediatr. Clin. North Am. 51, 507–527 (2004). 15062682 10.1016/S0031-3955(03)00214-1

[b3] Borra R. J. H. & Sorensen a. G. Incidental findings in brain MRI research: What do we owe our subjects? JACR J. Am. Coll. Radiol. 8, 848–852 (2011). 22137002 10.1016/j.jacr.2011.08.009

[b4] Vernooij M. W. *et al.* Incidental Findings on Brain MRI in the General Population. New Engl. J. Med. 135–137 (2007). doi: 10.1016/S0513-5117(08)79003-3 17978290

[b5] Launay S., Robert Y., Thomas D. & Devisme L. Irm cérébrale fœtale et ventriculomégalie. J Radiol 83, 723–730 (2002). 12149589

[b6] Tu T. *et al.* Imaging of Spontaneous Ventriculomegaly and Vascular Malformations in Wistar Rats: Implications for Preclinical Research. J Neuropathol Exp Neurol 73, 1152–1165 (2014). 25383642 10.1097/NEN.0000000000000140 PMC4232989

[b7] Gaser C., Nenadic I., Buchsbaum B. R. & Hazlett E. a. & Buchsbaum, M. S. Ventricular Enlargement in Schizophrenia Related to Volume Reduction of the Thalamus, Striatum, and Superior Temporal Cortex. Am. J. Psychiatry 161, 154–156 (2004). 14702264 10.1176/appi.ajp.161.1.154

[b8] Coley B. D. Caffey’s Pediatric Diagnostic Imaging. (Elsevier- Health Sciences Division, 2013).

[b9] Mampalam T. J., Harsh G. R., Tien R. D., Dillon W. P. & Wilson C. B. Unilateral hydrocephalus in adults. Surg. Neurol. 35, 14–19 (1991). 1983877 10.1016/0090-3019(91)90196-g

[b10] Leviton A. & Gilles F. Ventriculomegaly, delayed myelination, white matter hypoplasia, and ‘periventricular’ leukomalacia: How are they related? Pediatr. Neurol. 15, 127–136 (1996). 8888047 10.1016/0887-8994(96)00157-9

[b11] Leitner Y. *et al.* The neurocognitive outcome of mild isolated fetal ventriculomegaly verified by prenatal magnetic resonance imaging. Am. J. Obstet. Gynecol. 201, 215.e1–215.e6 (2009). 19527899 10.1016/j.ajog.2009.04.031

[b12] Khelfaoui M. *et al.* Loss of X-linked mental retardation gene oligophrenin1 in mice impairs spatial memory and leads to ventricular enlargement and dendritic spine immaturity. J. Neurosci. 27, 9439–9450 (2007). 17728457 10.1523/JNEUROSCI.2029-07.2007 PMC6673114

[b13] Mallard E. C., Rehn A., Rees S., Tolcos M. & Copolov D. Ventriculomegaly and reduced hippocampal volume following intrauterine growth-restriction: Implications for the aetiology of schizophrenia. Schizophr. Res. 40, 11–21 (1999). 10541002 10.1016/s0920-9964(99)00041-9

[b14] Ment L. R., Schwartz M., Makuch R. W. & Stewart W. B. Association of chronic sublethal hypoxia with ventriculomegaly in the developing rat brain. Dev. Brain Res. 111, 197–203 (1998). 9838111 10.1016/s0165-3806(98)00139-4

[b15] Roy P. D. *et al.* Temporal horn enlargement is present in schizophrenia and bipolar disorder. Biol. Psychiatry 44, 418–422 (1998). 9777171 10.1016/s0006-3223(98)00105-x

[b16] Hauser P. *et al.* MRI-based measurements of temporal lobe and ventricular structures in patients with bipolar I and bipolar II disorders. J. Affect. Disord. 60, 25–32 (2000). 10940444 10.1016/s0165-0327(99)00154-8

[b17] Apostolova L. *et al.* Hippocampal and ventricular changes in Parkinson’s disease mild cognitive impairment. Neurobiol. Aging 33, 2113–2124 (2012). 21813212 10.1016/j.neurobiolaging.2011.06.014 PMC4077346

[b18] Falip C. *et al.* Postnatal clinical and imaging follow-up of infants with prenatal isolated mild ventriculomegaly: A series of 101 cases. Pediatr. Radiol. 37, 981–989 (2007). 17724586 10.1007/s00247-007-0582-2

[b19] Simon H. & Newell A. Human problem solving: The state of the theory in 1970. Am. Psychol. 26, 145–159 (1970).

[b20] Harlow H. F. The formation of learning sets. Psychol. Rev. 56, 51–65 (1949). 18124807 10.1037/h0062474

[b21] Levine M. Hypothesis behavior. In: Schrier A.M., Harlow H.F., Stollnitz F. (Eds.), Behavior of Nonhuman Primates, vol. I. (1965).

[b22] MacDonald S. E., Pang J. C. & Gibeault S. Marmoset (Callithrix jacchus jacchus) spatial memory in a foraging task: win-stay versus win-shift strategies. J. Comp. Psychol. 108, 328–334 (1994). 7813192 10.1037/0735-7036.108.4.328

[b23] Duncan J. The multiple-demand (MD) system of the primate brain: mental programs for intelligent behaviour. Trends Cogn. Sci. 14, 172–179 (2010). 20171926 10.1016/j.tics.2010.01.004

[b24] Bugmann G., Goslin J. & Duchamp-Viret P. The speed of learning instructed stimulus-response association rules in human: Experimental data and model. Brain Res. 1536, 2–15 (2013). 23988509 10.1016/j.brainres.2013.07.046

[b25] Locurto C., Emidy C. & Hannan S. Mice (Mus musculus) learn a win-shift but not a win-stay contingency under water escape motivation. J. Comp. Psychol. 116, 308–312 (2002). 12234081 10.1037/0735-7036.116.3.308

[b26] Ainge J. A., Tamosiunaite M., Woergoetter F. & Dudchenko P. a. Hippocampal CA1 place cells encode intended destination on a maze with multiple choice points. J. Neurosci. 27, 9769–9779 (2007). 17804637 10.1523/JNEUROSCI.2011-07.2007 PMC6672960

[b27] Packard M. G., Hirsh R. & White N. M. Differential effects of fornix and caudate nucleus lesions on two radial maze tasks: evidence for multiple memory systems. J. Neurosci. 9, 1465–1472 (1989). 2723738 10.1523/JNEUROSCI.09-05-01465.1989 PMC6569845

[b28] Wise S. P. & Murray E. a. Role of the hippocampal system in conditional motor learning: Mapping antecedents to action. Hippocampus 9, 101–117 (1999). 10226772 10.1002/(SICI)1098-1063(1999)9:2<101::AID-HIPO3>3.0.CO;2-L

[b29] Bussey T.J., Wise S.P. & Murray E. A. The role of ventral and orbital prefrontal cortex in conditional visuomotor learning and strategy use in rhesus monkeys (Macaca mulatta). Behav. Neurosci. 115, 971–982 (2001). 11584930 10.1037//0735-7044.115.5.971

[b30] Collins P., Roberts A., Dias R., Everitt B. & Robbins T. Perseveration and strategy in a novel spatial self-ordered sequencing task for nonhuman primates: effects of excitotoxic lesions and dopamine depletions of the prefrontal cortex. J. Cogn. Neurosci. 10, 332–354 (1998). 9869708 10.1162/089892998562771

[b31] Gaffan D., Easton A. & Parker A. Interaction of inferior temporal cortex with frontal cortex and basal forebrain: double dissociation in strategy implementation and associative learning. J. Neurosci. 22, 7288–7296 (2002). 12177224 10.1523/JNEUROSCI.22-16-07288.2002 PMC6757896

[b32] Sakamoto T. & Okaichi H. Use of win-stay and win-shift strategies in place and cue tasks by medial caudate putamen (MCPu) lesioned rats. Neurobiol. Learn. Mem. 76, 192–208 (2001). 11502149 10.1006/nlme.2001.4006

[b33] Baxter M. G. & Murray E. A. The amygdala and reward. Nat. Rev. Neurosci. 3, (2002). 10.1038/nrn875 12094212

[b34] Cador M., Robbins T. W. & Everitt B. J. Involvement of the amygdala in stimulus-reward associations: interaction with the ventral striatum. Neuroscience 30, 77–86 (1989). 2664556 10.1016/0306-4522(89)90354-0

[b35] Kentridge R. W., Shaw C. & Aggleton J. P. Amygdaloid lesions and stimulus-reward associations in the rat. Behav. Brain Res. 42, 57–66 (1991). 2029345 10.1016/s0166-4328(05)80040-3

[b36] Holland P. C. & Gallagher M. Amygdala-frontal interactions and reward expectancy. Curr. Opin. Neurobiol. 14, 148–155 (2004). 15082318 10.1016/j.conb.2004.03.007

[b37] Rudebeck P. H., Mitz A. R., Chacko R. V. & Murray E. A. Effects of amygdala lesions on reward-value coding in orbital and medial prefrontal cortex. Neuron 80, 1519–1531 (2013). 24360550 10.1016/j.neuron.2013.09.036 PMC3872005

[b38] Apicella P., Ljungberg T., Scarnati E. & Schultz W. Responses to reward in monkey dorsal and ventral striatum. Exp. Brain Res. 85, 491–500 (1991). 1915708 10.1007/BF00231732

[b39] Delgado M. R. Reward-related responses in the human striatum. Ann. N. Y. Acad. Sci. 1104, 70–88 (2007). 17344522 10.1196/annals.1390.002

[b40] O’Doherty J. *et al.* Dissociable roles of ventral and dorsal striatum in instrumental conditioning. Science 304, 452–454 (2004). 15087550 10.1126/science.1094285

[b41] Robinson O. J., Frank M. J., Sahakian B. J. & Cools R. Dissociable responses to punishment in distinct striatal regions during reversal learning. Neuroimage 51, 1459–1467 (2010). 20303408 10.1016/j.neuroimage.2010.03.036 PMC3038262

[b42] Nakamura K. & Hikosaka O. Role of dopamine in the primate caudate nucleus in reward modulation of saccades. J. Neurosci. 26, 5360–5369 (2006). 16707788 10.1523/JNEUROSCI.4853-05.2006 PMC6675290

[b43] Yu R., Mobbs D., Seymour B. & Calder A. J. Insula and striatum mediate the default bias. J. Neurosci. 30, 14702–14707 (2010). 21048128 10.1523/JNEUROSCI.3772-10.2010 PMC6633627

[b44] Burke S. N. *et al.* Orbitofrontal Cortex Volume in Area 11/13 Predicts Reward Devaluation, But Not Reversal Learning Performance, in Young and Aged Monkeys. J. Neurosci. 34, 9905–9916 (2014). 25057193 10.1523/JNEUROSCI.3918-13.2014 PMC4107407

[b45] Rudebeck P. H. & Murray E. a. Dissociable effects of subtotal lesions within the macaque orbital prefrontal cortex on reward-guided behavior. J. Neurosci. 31, 10569–10578 (2011). 21775601 10.1523/JNEUROSCI.0091-11.2011 PMC3171204

[b46] Muranishi M. *et al.* Inactivation of the putamen selectively impairs reward history-based action selection. Exp. Brain Res. 209, 235–246 (2011). 21298425 10.1007/s00221-011-2545-y PMC3041916

[b47] Toates F. Biological Psychology. Animal Behaviour 82, (2011).

[b48] Skelin I. *et al.* Lesions of dorsal striatum eliminate lose-switch responding but not mixed-response strategies in rats. Eur. J. Neurosci. 39, 1655–1663 (2014). 24602013 10.1111/ejn.12518

[b49] Olton D. S. & Samuelson R. J. Remembrance of places passed: Spatial memory in rats. J. Exp. Psychol. Anim. Behav. Process. 2, 97–116 (1976).

[b50] Divac I., Rosvold H. E. & Szwarcbart M. K. Behavioral effects of selective ablation of the caudate nucleus. J. Comp. Physiol. Psychol. 63, 184–190 (1967). 4963561 10.1037/h0024348

[b51] Cohen S. M. Electrical stimulation of cortical-caudate pairs during delayed successive visual discrimination in monkeys. Acta Neurobiol. Exp. 32, 211–233 (1972). 4627615

[b52] Ellen P. & Wilson A. S. Perseveration in the rat following hippocampal lesions. Exp. Neurol. 8, 310–317 (1963).

[b53] Talpos J. C., Dias R., Bussey T. J. & Saksida L. M. Hippocampal lesions in rats impair learning and memory for locations on a touch-sensitive computer screen: The ‘ASAT’ task. Behav. Brain Res. 192, 216–225 (2008). 18499279 10.1016/j.bbr.2008.04.008

[b54] Yoon T., Okada J., Jung M. & Kim J. Prefrontal cortex and hippocampus subserve different components of working memory in rats. Learn. Mem. 15, 97–105 (2008). 18285468 10.1101/lm.850808 PMC2275661

[b55] Wang G.-W. & Cai J.-X. Disconnection of the hippocampal-prefrontal cortical circuits impairs spatial working memory performance in rats. Behav. Brain Res. 175, 329–336 (2006). 17045348 10.1016/j.bbr.2006.09.002

[b56] Cimadevilla J. M., Miranda R., López L. & Arias J. L. Partial unilateral inactivation of the dorsal hippocampus impairs spatial memory in the MWM. Cogn. Brain Res. 25, 741–746 (2005). 10.1016/j.cogbrainres.2005.09.001 16216479

[b57] Poucet B. & Buhot M. C. Effects of medial septal or unilateral hippocampal inactivations on reference and working spatial memory in rats. Hippocampus 4, 315–321 (1994). 7842055 10.1002/hipo.450040315

[b58] Tsujimoto S. & Sawaguchi T. Working memory of action: A comparative study of ability to selecting response based on previous action in New World monkeys (Saimiri sciureus and Callithrix jacchus). Behav. Processes 58, 149–155 (2002). 12044691 10.1016/s0376-6357(02)00041-4

[b59] Ahmed L. & de Fockert J. W. Focusing on Attention: The Effects of Working Memory Capacity and Load on Selective Attention. PLoS One 7, e43101 (2012). 22952636 10.1371/journal.pone.0043101 PMC3429456

[b60] Mikulka P. J. & Freeman F. G. The Effects of Reinforcement Delay and Hippocampal Lesions on the Acquisition of a Choice Response. Behav. Biol. 477, 473–477 (1975). 1212155 10.1016/s0091-6773(75)92264-6

[b61] Marzouki Y., Gullstrand J., Goujon A. & Fagot J. Baboons’ Response Speed Is Biased by Their Moods. PLoS One 9, e102562 (2014). 25061682 10.1371/journal.pone.0102562 PMC4111360

[b62] Yang S.-T., Shi Y., Wang Q., Peng J.-Y. & Li B.-M. Neuronal representation of working memory in the medial prefrontal cortex of rats. Mol. Brain 7, 61 (2014). 25159295 10.1186/s13041-014-0061-2 PMC4237901

[b63] Zhang X.-H., Liu S.-S., Yi F., Zhuo M. & Li B.-M. Delay-dependent impairment of spatial working memory with inhibition of NR2B-containing NMDA receptors in hippocampal CA1 region of rats. Mol. Brain 6, 13 (2013). 23497405 10.1186/1756-6606-6-13 PMC3616959

[b64] Brasted P. J., Bussey T. J., Murray E. A. & Wise S. P. Role of the hippocampal system in associative learning beyond the spatial domain. Brain 126, 1202–1223 (2003). 12690059 10.1093/brain/awg103

[b65] Banta Lavenex P., Amaral D. G. & Lavenex P. Hippocampal lesion prevents spatial relational learning in adult macaque monkeys. J. Neurosci. 26, 4546–4558 (2006). 16641234 10.1523/JNEUROSCI.5412-05.2006 PMC6674053

[b66] Tsao P. N., Teng R. J., Wu T. J., Yau K. I. T. & Wang P. J. Nonprogressive congenital unilateral ventriculomegaly. Pediatr. Neurol. 14, 66–68 (1996). 8652021 10.1016/0887-8994(95)00256-1

[b67] Tardif S.D., Power M.L., Ross C.N. & Rutherford J.N. Body Mass Growth in Common Marmosets: Toward a Model of Pediatric Obesity. Am. J. Phys. Anthropol. 150, 21–28 (2013). 23283661 10.1002/ajpa.22110 PMC3607500

[b68] Pessoa D. M., Tomaz C. & Pessoa V. Color Vision in Marmosets and Tamarins: Behavioral Evidence. Am. J. Primatol. 1222, 1210–1222 (2005). 10.1002/ajp.20202 16342075

[b69] Takemoto A., Izumi A., Miwa M. & Nakamura K. Development of a compact and general-purpose experimental apparatus with a touch-sensitive screen for use in evaluating cognitive functions in common marmosets. J. Neurosci. Methods 199, 82–86 (2011). 21549751 10.1016/j.jneumeth.2011.04.029

[b70] Hikishima K. *et al.* Population-averaged standard template brain atlas for the common marmoset (Callithrix jacchus). Neuroimage 54, 2741–2749 (2011). 21044887 10.1016/j.neuroimage.2010.10.061

[b71] Carpenter J. & Bithell J. Bootstrap confidence intervals: when, which, what? A practical guide for medical statisticians. Stat. Med. 19, 1141–1164 (2000). 10797513 10.1002/(sici)1097-0258(20000515)19:9<1141::aid-sim479>3.0.co;2-f

