# Supplementary material for: Cognitive impairment in a young marmoset reveals lateral ventriculomegaly and a mild hippocampal atrophy: a case report
Source: Sci Rep. 2015 Nov 3;5:16046. doi: 10.1038/srep16046 (PMC4630607; doi:10.1038/srep16046)
Supplement: Supplementary Information [file srep16046-s1.pdf]

Cognitive impairment in a young marmoset reveals lateral ventriculomegaly and a mild hippocampal atrophy: a case report

A. Sadoun[1, 2,*] ; K. Strelnikov[1, 2]; E. Bonté[1, 2]; C. Fonta[1, 2,#] ; P. Girard[1, 2,3,#]

Supplementary Methods

Animal housing

Animals were maintained in a temperature and humidity-controlled room (23-25°C and 66-68% humidity) and exposed to a 12:12 hours light-dark cycle. After the daily behavioural session, each animal was fed with a piece of gingerbread and pellets supplemented with portions of fruits and vegetables. In addition, 25ml of yogurt with vitamins and trace elements were given twice a week and the same quantity of mashed chickpeas once a week. Water was available continuously in the home cages.

Apparatus

Two exemplars of our experimental device were built in our laboratory. It consisted of a LCD infra-red touch screen (12.1", Winsonic Electronics CO., LTD). Transparent Plexiglas plates (28 x 26cm) with square holes (3,5 x 3,5 cm) were fixed at 1cm in front of the screen to avoid accidental body contact with the screen. These holes allowed the animals to touch the screen at the exact location where the visual gray-level stimulus appeared. In addition, a mini camera was placed at the side of the apparatus to monitor the behaviour of the animal. These components were fixed inside a plastic black box (52 x 31 x 11 cm) and each experimental device was connected to a PC (Dell Core2duo) running the software (Matlab. R2011a).

MRI automatic segmentation methods

Automatic segmentation was performed in each slice in a square of 42 x 63 pixels, which was chosen at the same location in each animal and comprised the hippocampus and the adjacent ventricle. We used the automatic segmentation tool Atropos, an open source segmentation algorithm provided with ANTs, which is an ITK-based C++ package (www.picsl.upenn.edu/ANTs), ([1]). This algorithm has a Bayesian component and requires template images as priors. Three binary priors were manually created on the basis of the structures at the same location in the template: the right hippocampus prior, the cerebrospinal fluid prior and the white matter prior. We applied the Matlab Gaussian smoothing filter of 2 mm to the Template priors.

Before the automatic segmentation, we adjusted the parameters of the Atropos method in one animal to obtain optimal fitting of the resulting binary masks to the hippocampus in this animal. The chosen parameters were then applied to all the other animals including the PathC. We chose the default value of the smoothing factor (0.3) for the Markov random field. The prior probability images were used only for initialisation with the value=0; the Convergence criteria was 1.e-5 ([1]). The whole command line being like this:

```
Atropos -a 111_Square.tiff -c [5,0.00001] -m [0.3,1] -u 0 -x
mask2D.tiff -i PriorProbabilityImages[3,111_2D_%%02d.tiff, 0] -o
[111_labels.nii, 111_nposterior_%%02d.nii]
```


1. Avants, B. B., Tustison, N. J. & Gee, J. C. Segmentation with Evaluation on Public Data. *Neuroinformatics* **9,** 381–400 (2012).
